# Cardiorespiratory Effects of Three Infusion Doses of Adenosine in Conscious Goats: A Preliminary Study

**DOI:** 10.3390/vetsci8080158

**Published:** 2021-08-06

**Authors:** Eman Salah, Mahmoud M. Abouelfetouh, Ryane E. Englar, Mingxing Ding, Yi Ding

**Affiliations:** 1 College of Veterinary Medicine, Huazhong Agricultural University, Wuhan 430070, China; eman.salah@fvtm.bu.edu.eg (E.S.); mahmoud.abouelfetouh@fvtm.bu.edu.eg (M.M.A.); dmx@mail.hzau.edu.cn (M.D.); 2 Department of Pharmacology, College of Veterinary Medicine, Benha University, Moshtohor, Toukh 13736, Egypt; 3 Department of Surgery, Radiology and Anaesthesiology, College of Veterinary Medicine, Benha University, Moshtohor, Toukh 13736, Egypt; 4 College of Veterinary Medicine, University of Arizona, Oro Valley, AZ 85737, USA; renglar@arizona.edu

**Keywords:** adenosine, adenosine infusion, cardiorespiratory parameters, goats

## Abstract

Adenosine (AD) has been implicated in human healthcare as an endogenous signaling nucleotide in both physiologic and pathologic states. The effects of AD on cardiorespiratory parameters in ruminants has not yet been studied. The objective of this study was to evaluate the cardiac and respiratory changes that resulted from an intravenous AD infusion in goats. Six clinically healthy adult goats weighing 28 ± 2 kg were randomly assigned to one of four treatments in a crossover design with a seven day washout period. The goats received a 0.9 % saline solution (SAL treatment) and three AD treatments (AD 50, 100 and 200) intravenously at a dose rate of 50, 100 and 200 μg/kg/min. Cardiorespiratory and key cardiac parameters were measured before the treatment (baseline), during the infusion (dInf) and at 1, 3, 5 and 10 min after each infusion was discontinued. The AD 100 produced a significant increase in HR (*p* = 0.001) and the AD 200 resulted in significant rises in HR (*p* = 0.006) and RR (*p* = 0.001) compared with the baseline. This study concluded that the AD infusion could trigger an increase in HR and RR in a dose-dependent manner in healthy goats.

## 1. Introduction

Adenosine (AD) is a purine nucleoside cell signaling agent that exerts its biological effects via four distinct receptors (A_1_, A_2A_, A_2B,_ A_3_). It has a short half-life ranging from seconds to minutes [1]. AD receptors play a pivotal regulatory role not only in normal cell biology and physiology but also in pathologic states. The binding of AD to A_1_ receptors inhibits the catecholamine release and slows the atrioventricular conduction in the heart [2]. Additionally, AD promotes vasodilation in all but renal vasculature [3,4,5].

Not all AD receptors appear to exert the same physiologic effects. The binding of AD to A_2A_ receptors indirectly increases cardiac contractility in humans by modulating the antiadrenergic effect that AD has when it binds to A_1_ [6]. Recent reports within the human medical literature have confirmed that AD has the potential to promote a sympathetic outflow and chemoreceptor activation through this mechanism [7,8,9,10,11].

Goats are increasingly raised as companion animals and maintaining their cardiovascular health is an important part of preventative care. Several cardiac arrhythmias such as sinus tachycardia and respiratory sinus arrythmia have been reported in goats and sheep [12]. In addition, supraventricular (SVT) and ventricular tachycardias have been induced by bupivacaine, a commonly used local anesthetic in small ruminants [13].

AD has been increasingly used in human clinical trials as a diagnostic and antiarrhythmic agent. In patients with paroxysmal SVT, AD could slow down atrioventricular (AV) nodal conduction by interrupting the electrical impulse pathways, thereby converting SVT to a normal sinus rhythm [14,15,16]. The transient AV block that results may be also helpful for diagnosing complex tachycardias such as atrial flutter and atrial fibrillation. AD may also be a safe and effective agent that could be used to induce hypotensive states in those surgical interventions that require it, for example, orthopedic and cerebral aneurysm surgeries [17,18].

Pain management is also a critical part of healthcare in any veterinary practice, including those that cater for small ruminants. When AD binds to A_1_ receptors, neural activity is reduced and the pain threshold in the spinal cord is raised [19]. This raises pain tolerance in veterinary patients. The binding of AD to A_1_ also synergistically enhances the antinociceptive effect of clonidine [20] and acupuncture [21].

AD has also been investigated as a potential anti-inflammatory agent with clinical relevance in managing ischemia-reperfusion injuries and septic shock [22]. It appears that AD may be cardioprotective in these clinical scenarios by promoting vasodilation, reducing inflammation and inhibiting clotting. For this reason, AD A_2A_ agonists might be an alternative therapy for ischemia as well as hypertension-induced heart failure [23]. Despite these applications for the use of AD in human healthcare, veterinarians have not fully investigated the therapeutic and diagnostic potential of AD in clinical practice. The effects of AD on canine cardiovascular health have only recently been explored [24]. To the authors’ knowledge, the effects of an intravenous infusion of AD in ruminants remain unknown. The objective of this preliminary study was to investigate the cardiorespiratory effects of intravenous AD at three different infusion doses in healthy, conscious goats. We hypothesized that AD produces dose-dependent chronotropic effects in caprine patients.

## 2. Materials and Methods

### 2.1. Animals

Six clinically healthy goats ranging from 13–15 months of age and a body weight of 28 ± 2 kg were enrolled in this study. For the purpose of this study, the animals were considered physically fit as determined by a comprehensive physical examination (including cardiothoracic auscultation, ECG, echocardiography and testing within the normal reference ranges for packed cell volume (PCV), complete blood count (CBC) and serum biochemical profiles).

The six goats enrolled in this study were selected from a group of thirty goats based upon their handleability. The group was purchased locally and brought into an experimental research unit one week prior to the infusions for daily acclimatization to handling and the environmental design for a duration of thirty minutes per day. This study was approved by the animal experimental ethical inspection of the Laboratory Animal Center, Huazhong Agricultural University (HZAUGO-2019-004).

### 2.2. Study Design

**Figure 1:**
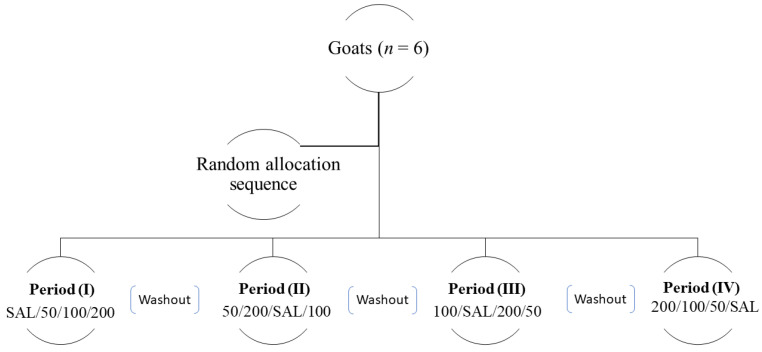
Different treatment sequence in the randomized crossover design. Each goat received saline (SAL) and adenosine at a dose rate of 50, 100 or 200 µg/kg/min but in a random sequence with a washout period of 7 days.

This experimental study used a prospective, randomized crossover design with a seven day washout period. Six adult goats were randomly assigned to one of four treatments using a computer program (www.randomizer.org) (accessed on 11 October 2019) in which all goats received the four treatments in a random order (Figure 1).

All goats were shaved in the distal one third region between the 3rd and 6th intercostal spaces on their right thoraxes and placed with minimum restraint on right lateral recumbency in preparation for echocardiography. The goat’s tail was shaved for the placing of a pulse oximeter probe. A 20-gauge 2.5 cm catheter was then placed in each goat’s left jugular vein in preparation for their infusion.

The animals received a 0.9 % saline solution (SAL treatment) and three AD (Adenocor^®^ 3 mg/mL; Sanofi; Berkshire, UK) treatments (AD 50, 100 and 200) at a dose rate of 50, 100 and 200 μg/kg/min for 2 min using a syringe driver through their jugular catheter. There was a 1 min stabilization period before taking measurements after starting the infusion. AD was diluted to reach a concentration of 600 mg/mL so that the total volume infused ranged from 2 to 10 mL. Identical volumes of saline were infused in the SAL treatment.

**Figure 2:**
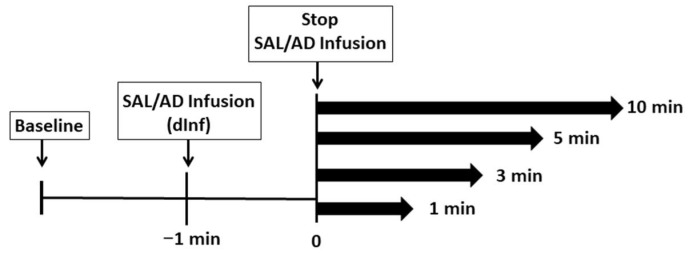
Study timeline showing electrocardiogram (ECG) recordings and echocardiographic measurements in goats receiving saline (SAL) and three adenosine (AD) doses at the baseline, during the infusion (dInf) and at 1, 3, 5 and 10 min after discontinuing the infusion.

Cardiorespiratory and key echocardiographic parameters were measured before the treatment (baseline), during the infusion (dInf) and at 1, 3, 5 and 10 min after discontinuing each infusion (Figure 2).

#### 2.2.1. Cardiorespiratory Parameters and Rectal Temperature

A multi-parameter 3-lead electrocardiogram patient monitor (Mindray MEC-1200 Vet, Mahwah, NJ, USA) was used to measure HR, beats/min, hemoglobin oxygen saturation (SpO_2_, %) and respiratory rate (RR, breaths/min) as well as the rectal temperature (RT, °C). The reflective pulse oximeter probe was placed over the goat’s shaved tail.

#### 2.2.2. Echocardiographic Parameters

Guided M-Mode 2D echocardiography was conducted using an ultrasound device (Siemens, X-300, Seongnam, Korea). Cardiac imaging was obtained using the right parasternal long axis views. To minimize imprecision, overestimation and intra-observer variability, an experienced cardiac sonographer was tasked with gathering all images. To evaluate the reliability and consistency of the examiner, the intra-observer variability (measurement error) was assessed ([mean difference between measurements/average of measurements] × 100) and expressed as the coefficient of variation in a percent (%). Intra-observer variability was valued by three repeated measurements of the same M-mode image performed by the examiner in six goats. The cardiac scanning technique and linear measurement of the left ventricular internal dimensions during diastole (LVIDd) and systole (LVIDs), the interventricular septum dimension (IVSD) and the left ventricular posterior wall dimension (LVPWD) were standardized in line with recommendations from the American Society of Echocardiography and the European Association of Cardiovascular Imaging [25]. The long axis views were obtained at the mitral valve level. Angled and oblique views were avoided. The measurement values were obtained carefully perpendicular to the left ventricle long axis at the level of the mitral valve leaflet tip. The calibration of the LV internal dimensions was carried out using ultrasound integrated software, which automatically computes the ejection fraction (EF) (%), fractional shortening (FS) (%) and stroke volume (mL). CO (L/min) was calculated by multiplying the ECG-recorded HR by the stroke volume (SV); thus,

CO = HR×SV.

### 2.3. Statistical Analysis

Statistical tests were performed using GraphPad Prism software version 8.0 (GraphPad Inc, San Diego, CA, USA). Continuous data of cardiorespiratory and echocardiographic variables were taken at the baseline, during the infusion (dInf) and at 1, 3, 5 and 10 min after discontinuing the AD infusion. All data were expressed as mean ± the standard deviation (SD). After confirming a normal distribution of data using the Kolmogorov–Smirnov test, the analysis of variance (ANOVA) with Dunnett’s test post-hoc was used to compare each group with the baseline and also between AD and SAL treatments. The differences were considered significant at *p* < 0.05.

## 3. Results

**Figure 3:**
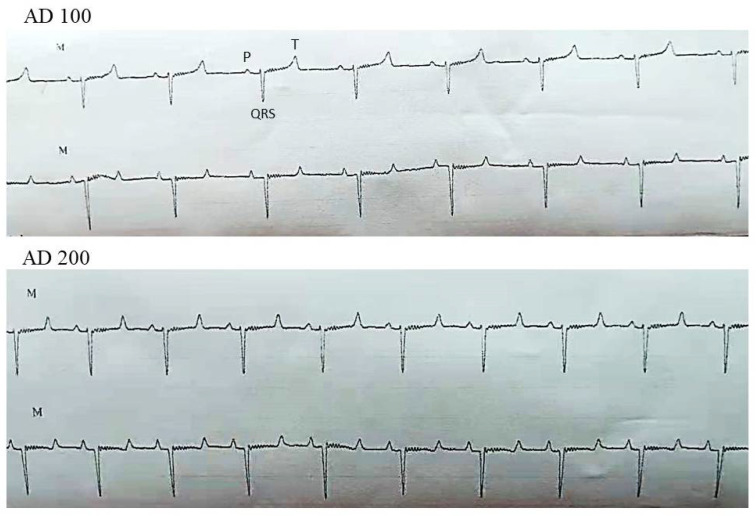
Lead II electrocardiograms of the AD 100 and 200 showing no signs of atrial or ventricular arrythmia during the AD infusion. Note the normal shape of the P and QRS wave with a regular rhythm.

There were no significant differences in the baseline values of HR, RR, SpO_2_ and RT between the SAL and the three AD treatments. During the infusion (dInf), the AD 100 and 200 showed an increase in HR; however, only the AD 200 exhibited a significant rise in HR compared with the SAL (*p* = 0.003). At the one minute mark following the discontinuation of the infusion, HR rapidly reduced; however, it significantly increased in the AD 200 compared with the SAL (*p* < 0.001). Compared with the baseline, HR did not change in the SAL and the AD 50 but increased significantly in the AD 100 (*p* = 0.001) and 200 (*p* = 0.006) at dInf. At the one minute mark following the discontinuation of the infusion, HR returned to the normal range in the AD 100 but remained significantly higher in the AD 200 (*p* = 0.035). Moreover, the RR increased significantly in the AD 200 (*p* < 0.001) at dInf. However, AD 50 and 100 did not exhibit a significant difference compared with the baseline. The AD infusions did not influence the baseline values of SpO_2_ and RT (Table 1). In all treatments, non-significant variations occurred in HR, RR, SpO_2_ and RT at 3, 5 and 10 min after discontinuing the infusion. No signs of arrythmia and abnormalities in the QRS and P wave observed in the electrocardiograms in all AD treatments (Figure 3).

In this study, EF, FS and CO were measured as vital indicators for the left ventricular function. Compared with the SAL treatment, all AD treatments did not show significant differences at dInf and at 1, 3, 5 and 10 min after discontinuing the infusion. Even though HR significantly increased at dInf in the AD 100 and 200, the AD infusion did not significantly alter the baseline values for the cardiac parameters within each treatment (Table 2).

## 4. Discussion

This preliminary study indicated that intravenous infusions of AD in conscious goats produce a transient, dose-dependent, excitatory effect on HR and RR. In humans, AD infusion at a dose rate of 50 and 100 μg/kg/min has been reported to induce positive chronotropy with a non-significant change in systemic blood pressure [26,27]. Another study found that the steady state infusions of AD at a dose rate of 140 µg/kg/min resulted in a significant increase in HR (+30 beats/min) [7]. In this current study, AD infusion in goats produced a modification in HR and RR resembling that occurring in humans. In pregnant ewes, the intravenous injection of AD at a dose of 200 μg/kg was also reported to produce an acceleration in HR [28]. The hemodynamic effect of AD is dependent on its dose, route of administration and if the patient is conscious or anesthetized. Non-significant changes in HR, CO and cardiac index were found during a continuous AD infusion at a dose rate of 140 or 280 μg/kg/min in anesthetized dogs [24]. Moreover, minor increases in HR (+9 beats/min) and CO (+2 L/min) were observed with an AD infusion of 0.14 ± 0.04 mg/kg/min in patients undergoing cerebral aneurysm surgery [29]. In contrast to its effects in the anesthetized state, AD infused at a dose rate of 140 μg/kg/min in conscious volunteers initiated a significant rise in HR, thoracic excursion (+200%), partial arterial oxygen pressure (+12 mmHg) and a decrease in partial arterial carbon dioxide pressure (−10 mmHg) [7]. In another study, intravenous boluses of AD at four different doses (80, 100, 120 and 140 µg/kg) resulted in significant increases in both the depth and rate of respiration [30].The effect of an AD infusion seems to be less excitatory and blunted with anesthesia, which may be due to the sympatholysis induced by the anesthetic agents.

In the present study, HR increased in a dose-dependent manner during AD infusion in which a higher significant increase was observed in the AD 200 as opposed to the AD 100. However, the AD 50 showed no effect on the baseline values. Our findings were consistent with prior results found during an AD infusion in conscious humans [7,31]. Furthermore, conscious dogs showed a significant increase in HR and a non-significant alteration in CO following injections of AD at doses of 10, 25, 50, 100 and 250 μg/kg [32]. AD has been well-known to induce cardiac depression and hypotension via the activation of AD A_1_, resulting in the vasodilation of most vascular beds in the body [33]. In this study, the AD-induced tachycardia might be a compensatory reflex caused by hypotension. Moreover, the autonomic innervation and vagal tone of the heart might play a pivotal role in the AD-induced tachycardia because the increase in HR induced by the AD infusion could be completely blocked in patients with an autonomic dysfunction [7]. Additionally, a bilateral vagotomy in normal conscious dogs could prevent the tachycardic response to the AD infusion [34]. The AD infusions were also associated with a dose-dependent increase in the plasma levels of norepinephrine and epinephrine [35]. The activation of a sympathetic outflow is mostly attributable to the AD-induced increases in HR in goats [8,10]. Carotid body chemoreceptor activation could be also induced by AD infusion, which may be involved in the respiratory stimulation [7]. Pulmonary stretch receptors could be activated due to hyperventilation and they tend to produce tachycardia [36]. In this current study, the increase in HR was observed during AD infusion, particularly with AD 200; however, non-significant changes occurred in CO, EF and FS. A decrease in SV was more likely to be the compensatory mechanism of maintaining CO. This finding was supported by a prior study in which a significant increase in HR with no change in CO was associated with intravenous boluses of AD up to 250 µg/kg [32]. The increase in HR may contribute to a decrease in end diastolic volume (EDV) due to the reduced time for ventricular filling during diastole. Another study revealed that an intravenous infusion of AD at a dose rate of 140 µg/kg/min produces a significant increase in HR (+18 beats/min) and a decrease in EDV (−9 mL) with a non-significant alteration in EF [37]. The small sample size in this study may have limited the value of the observations as a true representation of the goat population. In addition, the use of direct arterial blood pressure monitoring and arterial blood gas analysis may have provided a superior assessment of the effect of the AD infusion on the cardiopulmonary status.

## 5. Conclusions

In conscious goats, AD produced a dose-dependent increase in HR and RR without altering EF, FS and CO. The higher dose of AD could be used therapeutically as a cardiorespiratory stimulant. Although this preliminary study could provide valuable information about the effect of AD in conscious goats, a more comprehensive study is needed to evaluate the effect of AD in anesthetized animals as well as those with a cardiac compromise.

## Figures and Tables

**Table 1:** Cardiorespiratory variables and rectal temperature of goats (*n* = 6) receiving saline (SAL) and three adenosine (AD) dose infusions at the baseline (B), during the infusion (dInf) and at 1, 3, 5 and 10 min after discontinuing the infusion.

Parameters	Group	Time Points (Minutes)					
		B	dInf	1	3	5	10
HR (beats/min)	SAL	108.3 ± 5.2	98.1 ±14.2	91.5 ± 10.5	98.5 ± 10.4	109 ± 11.6	100.5 ± 12.5
	AD 50	108.7 ± 6.8	98.1 ± 6.8	101.8 ± 11.6	99.3 ± 9.1	102.3 ± 11	93.1 ± 6.9
	AD 100	103.3 ± 6	113.7 ± 4.2	94.6 ± 9.1	97.3 ± 9.9	99.6 ± 10.3	96.5 ± 13
			*p* = 0.001 ٭				
	AD 200	105.3 ± 8.6	131 ± 7.1 ٭	121.8 ± 6.4 ٭	106 ± 7	102 ± 7.8	105.2 ± 12.2
			*p* = 0.006	*p* = 0.035			
RR (breaths/min)	SAL	20.1 ± 3.7	21.5 ± 2.8	21 ± 5.8	22 ± 5.1	22.5 ± 4	21.3 ± 3.5
	AD 50	23.8 ± 3	22.6 ± 4.1	20 ± 3.2	21.6 ± 2.9	22.5 ± 4.4	21.3 ± 5.2
	AD 100	23 ± 5	22.1 ± 3.6	20.1 ± 2	20.6 ± 4.2	22.8 ± 4.9	23.8 ± 2
	AD 200	22.5 ± 3.3	41.1 ± 3٭	22.8 ± 3	21 ± 3	20.5 ± 3.5	23.3 ± 3
			*p* < 0.001				
SpO_2_ (%)	SAL	94.8 ± 3	93.8 ± 2.2	94 ± 2	93.5 ± 2.5	93.6 ± 2	94 ± 2.6
	AD 50	95.2 ± 3	94 ± 2	93.8 ± 2.9	94 ± 2.8	94 ± 2.6	94.6 ± 2.6
	AD 100	95.1 ± 3.2	95.3 ± 3	94.6 ± 3.6	95 ± 2.9	94.6 ± 3.5	94.5 ± 3.4
	AD 200	95.2 ± 2.9	94.8 ± 2.4	95.3 ± 2.8	95 ± 3.4	94.2 ± 2.5	94.1 ± 1.6
RT (°C)	SAL	39.4 ± 0.16	39.5 ± 0.29	39.5 ± 0.19	39.5 ± 0.17	39.5 ± 0.24	39.15 ± 0.19
	AD 50	39.4 ± 0.23	39.5 ± 0.21	39.5 ± 0.27	39.4 ± 0.23	39.5 ± 0.13	39.5 ± 0.10
	AD 100	39.4 ± 0.22	39.4 ± 0.24	39.5 ± 0.15	39.4 ± 0.27	39.4 ± 0.14	39.6 ± 0.12
	AD 200	39.5 ± 0.24	39.4 ± 0.17	39.5 ± 0.24	39.4 ± 0.30	39.5 ± 0.21	39.6 ± 0.20

HR, heart rate; RR, respiratory rate; SpO_2_, hemoglobin oxygen saturation; RT, rectal temperature. All data are presented as a mean ± SD. ٭ significantly different from B (*p* < 0.05).

**Table 2:** Echocardiographic variables of goats (*n* = 6) receiving saline (SAL) and three adenosine (AD) dose infusions at the baseline (B), during the infusion (dInf) and at 1, 3, 5 and 10 min after discontinuing the infusion.

Parameters	Group	Time Points (Minutes)					
		B	dInf	1	3	5	10
EF (%)	SAL	72.1 ± 5.9	71.8 ± 4.2	72.6 ± 5.7	71.6 ± 4.8	75.5 ± 4.4	74.5 ± 4
	AD 50	71.5 ± 2.8	72.8 ± 2.4	72.1 ± 4.8	71.8 ± 3.4	73 ± 2.9	73.5 ± 3
	AD 100	73 ± 4	74 ± 3.8	74.3 ± 3.3	73.5 ± 3.2	72.8 ± 4.7	72.5 ± 3.7
	AD 200	72.8 ± 2.7	75 ± 3.3	73.6 ± 4.2	73 ± 4.1	72.8 ± 4.4	73.8 ± 3.9
FS (%)	SAL	38.5 ± 3	39.3 ± 3.2	37.3 ± 4.6	38.3 ± 3.5	36.5 ± 2.5	38.1 ± 4
	AD 50	38.3 ± 2.7	37.6 ± 2.2	37.3 ± 2.7	36.6 ± 2.5	37.3 ± 2.1	36.8 ± 2.5
	AD 100	38 ± 3	38.3 ± 1.6	36.8 ± 1.4	37.3 ± 2.8	37 ± 2.6	36.6 ± 1.8
	AD 200	37 ± 2.3	38.3 ± 2.3	37 ± 2	36.6 ± 2.2	37 ± 1.7	36.5 ± 2
CO (L/min)	SAL	2.5 ± 0.3	2.3 ± 0.5	2.6 ± 0.3	2.4 ± 0.3	2.5 ± 0.4	2.6 ± 0.2
	AD 50	2.4 ± 0.28	2.4 ± 0.29	2.3 ± 0.33	2.3 ± 0.23	2.3 ± 0.30	2.5 ± 0.27
	AD 100	2.4 ± 0.36	2.5 ± 0.35	2.3 ± 0.32	2.2 ± 0.31	2.2 ± 0.21	2.3 ± 0.30
	AD 200	2.4 ± 0.35	2.5 ± 0.42	2.2 ± 0.32	2.1 ± 0.30	2.2 ± 0.21	2.3 ± 0.29

EF, ejection fraction; FS, fraction shortening; CO, cardiac output.

## Data Availability

The data presented in this study are available on request from the corresponding author.
